# Stepwise low concentration atropine for myopic control: a 10-year cohort study

**DOI:** 10.1038/s41598-021-96698-6

**Published:** 2021-08-30

**Authors:** Meng-Ni Chuang, Po-Chiung Fang, Pei-Chang Wu

**Affiliations:** Department of Ophthalmology, Kaohsiung Chang Gung Memorial Hospital and Chang Gung University College of Medicine, 123, Da-Pi Road, Niao-Sung District, Kaohsiung, 88301 Taiwan, R.O.C.

**Keywords:** Paediatrics, Eye diseases, Paediatric research, Eye manifestations

## Abstract

The aim of this study was to analyze changes in refraction and evaluate the variables in school children who received atropine as myopic control for 10 years. Low-concentration atropine (0.05%) was prescribed initially, and the dose was increased in a stepwise manner if rapid myopic progression (≥ 0.5D per half year) was noted during the regular follow-up visit. 23 children with a mean age of 6.96 ± 1.07 years were included. The initial spherical equivalent was − 1.25 ± 0.84 D. The overall mean myopic progression was − 0.30 ± 0.27 D/year. Younger initial age, female, higher initial spherical equivalent and the need of higher concentration of atropine were found to be risk factors for myopic progression in multivariate mixed-effect analysis (*p* = 0.013, 0.017, 0.024 and 0.014). Children who kept using a lower concentration of atropine (≤ 0.1%) tended to have slower myopic progression throughout the 10-year course than those who shifted to higher concentrations (> 0.1%) (*p* ≤ 0.001). Stepwise low concentration of atropine might be effective for long-term myopic control in school students. Those who had poor response to lower concentration of atropine may have the risk of faster progression, even with high concentration of atropine. Additional or alternative treatment might be considered.

Myopia is considered a global threat to vision and a rising public health issue. The worldwide prevalence of myopia and high myopia will increase to a proportion of 50% and 10%, respectively, which is equal to nearly 5 billion and 1 billion people by 2050^1^. The prevalence of myopia is noteworthy in East Asia. In Taiwan, Korea, Singapore, and Hong Kong, more than 80% of the young population has myopia, according to previous reports^2–5^. In non-Asian society, European Eye Epidemiology revealed a prevalence of 30% of middle-aged adults collected from 1990 to 2013^6^. As for the United States, an increasing trend has been reported, with the prevalence almost doubling during the past three decades from 25 to 40%^6^. Epidemiology studies from different nations all point out that this “myopia boom” should be managed seriously^7^.

Pathologic myopia, defined as “myopic-related, excessive axial elongation, which causes a structural change in the posterior segment of the eye,” is frequently associated with the development of high myopia^8^. Complications include retinal detachment, myopic maculopathy, macular retinoschisis, and myopic optic neuropathy^9^. If myopia begins earlier, with a longer duration and rapid rate of myopic progression, a higher risk of having high myopia can be assumed^10,11^. With an irreversible axial length increase, their vision in the upcoming years is threatened. According to a study conducted in the Netherlands, by the age of 75 years, the cumulative risk of visual impairment (best-corrected visual acuity ≤ 0.3) was as high as 39% in high myopic group, compared to only 3.8% in non-high myopic group^12^.

Knowing how severe myopia could damage one’s vision, strategies were surveyed in order to prevent the rapid progression of myopia. Among them, atropine plays an important role in myopic control^13^. Although high concentrations were shown to control myopia efficiently, many patients were unwilling to maintain this management due to prominent photophobia^14^. Low concentration atropine offers a new opportunity for myopic children. The atropine for the treatment of childhood myopia 2 (ATOM 2) study disclosed the efficacy of 0.01% atropine, along with minimal side effects during a 2-year study period^15^. The 2-year result of the low-dose atropine for myopic progression (LAMP) study found that 0.05% atropine had a better effect compared with 0.025% and 0.01% ones^16,17^. Nonetheless, myopic progression and subsequent axial length elongation lasts through the childhood and teenage years, which is much longer than one or two years. One study performed in Spain found that 0.01% atropine could reduce the myopic progression by 25% during a 5-year follow-up period^18^. For a longer trial, only one study in the United States applied high-dose atropine (1%) for a varied duration, ranging from 6 to 15 years^19^. To the best of our knowledge, a longer term evaluation of low concentration atropine in myopic control is necessary. This study aimed to evaluate the long-term outcome of myopia control by stepwise low-concentration atropine use.

## Methods

Participants were included based on the following criteria: children whose spherical equivalent at initial visit was ≤  − 0.5 D and had been regularly followed for ten years with an interval of 2–4 months, mostly 3 months between each visit during this period. Subjects with the following conditions were excluded from this study: (1) high astigmatism, ≥ 2.0 D, (2) congenital myopia, defined as SE ≤  − 5.0 D in children younger than six years old, (3) cases with other ocular diseases, such as amblyopia, strabismus, congenital glaucoma, congenital cataract, and ocular tumor according to chart review and ocular examination at every ophthalmic outpatient department visit, and (4) children who had been using or tried orthokeratology during the treatment course. The chart review started from January, 1999, till December, 2017. The study was approved by the Institutional Review Board of Chang Gung Memorial Hospital and adhered to the tenets of the Declaration of Helsinki. The IRB approves the waiver of the participant's consent.

**Figure 1:**
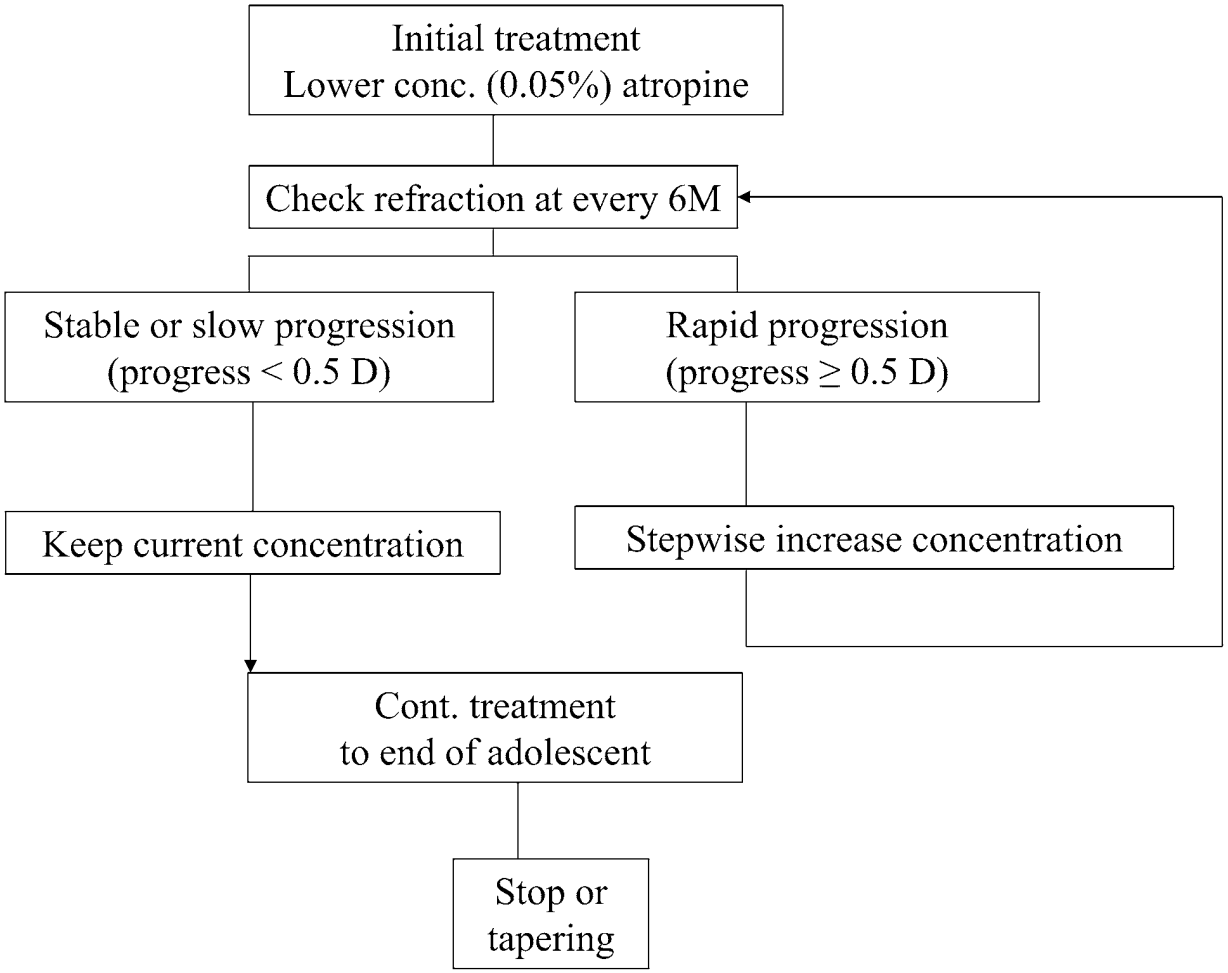
Protocol of stepwise low concentration of atropine treatment.

A stepwise strategy of atropine use in myopic control was applied to all subjects. Initially, a single drop of atropine with a concentration of 0.05% was applied every night. If the progression of myopia exceeded − 0.5 D in six months was considered as rapid progression, the concentration of atropine was stepwise increased. The amount of dose adjustment varied in different case individualized (Fig. 1). The concentrations of atropine were 0.05%, 0.1%, 0.25%, and 0.5%. All subjects in this study received atropine concentrations lower than 1%. In the late adolescent period, the concentration was decreased stepwise, especially in who had stable myopia control. The way to achieve these concentrations was by diluting 0.25%, 0.5%, and 1% atropine ophthalmic solutions (Atropine Sulphate Eye Drops; Wu-Fu Lab. Co., Ilan, Taiwan) with distilled water.

At the first visit, a thorough ocular examination was performed, including visual acuity, slit-lamp examination, and fundoscopy in order to identify other ocular abnormalities before starting treatment. On every regular visit to our ophthalmic outpatient department, cycloplegic refraction was adopted to obtain the refractive error. Cyclopentolate (1%) was applied once, followed by instillation of 1% tropicamide twice at a 10-min interval. During the follow-up years, an autorefractor (KR-7000/8100/8900/800; Topcon, Tokyo, Japan) was used to measure SE and regularly calibrated. The patients were only prescribed single vision lenses if they had the need of correction. They did not receive the prescription of progressive addition lenses (PAL) or other peripheral correction interventions.

For homogenous comparison of eye refraction data, analyses were conducted on the SER of the right eye only, because refractive error was highly correlated between the right and left eyes (r = 0.92, *p* < 0.001). The software used was the Statistical Package for the Social Sciences software (SPSS version 22, Chicago, IL). Descriptive analysis was applied to calculate overall myopia progression rate. To determine if sex, initial age, initial SE and concentration of atropine would be associated with myopic progression, mixed-effect regression model was used. In addition, independent t-test were used to determine the difference between the stepwise lower concentration atropine group (≤ 0.1%) and the stepwise higher concentration group, which was defined as those who had ever received a dose higher than 0.1% of atropine. Clinical significance was defined as *p* value < 0.05.

## Results

**Figure 2:**
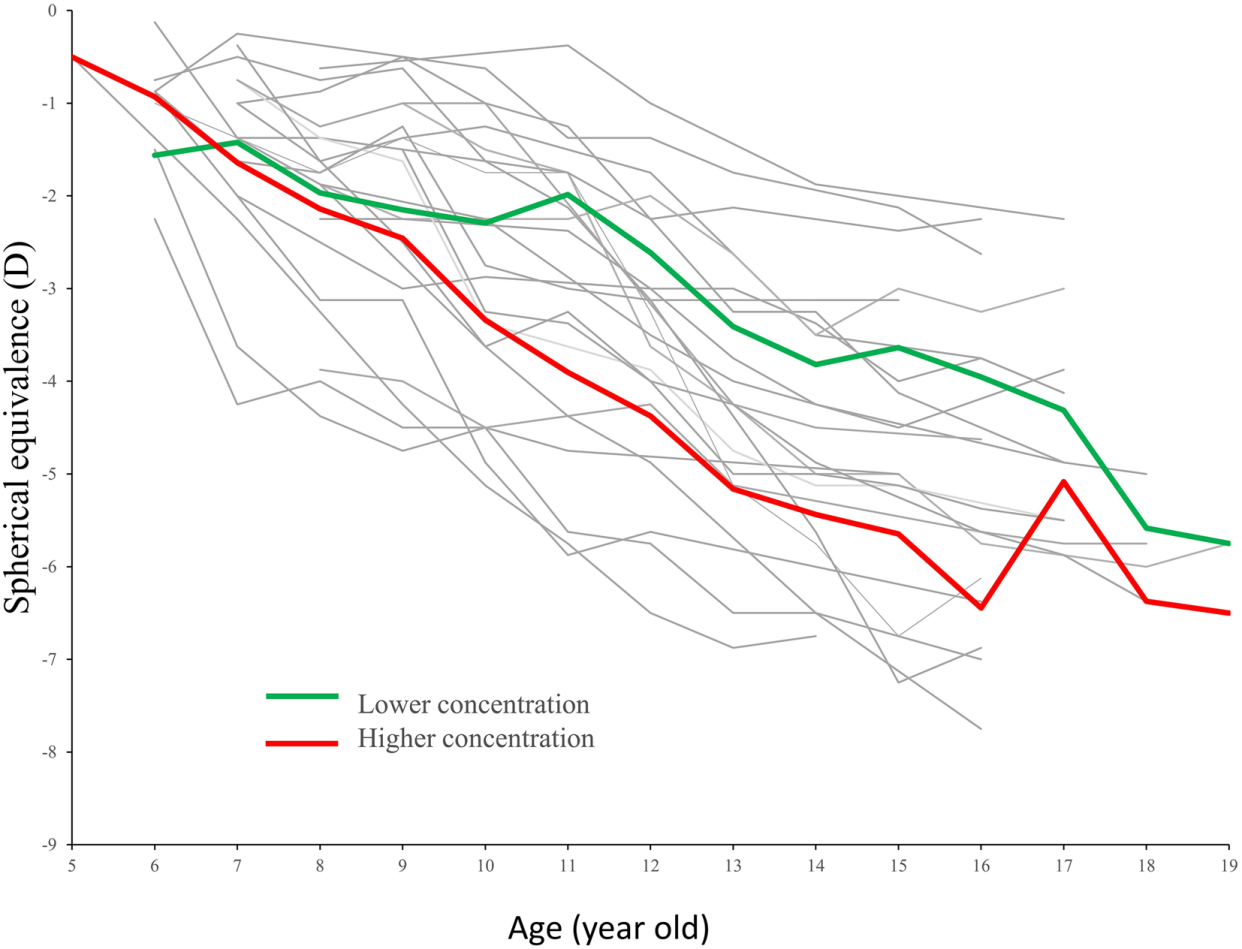
Spherical equivalence and the age of each individual subject.

A total of 23 subjects who had been regularly followed-up at our ophthalmic outpatient department were included in the study. Among them, 9 cases were male (39.1%). The mean initial age was 6.96 ± 1.07 years, ranging from 5 to 9 years. The initial SE after cycloplegia was − 1.25 ± 0.84 D. Figure 2 demonstrated the progression of SE and the age of individual case. During this 10-year period, 65% of patients kept treatment with lower concentration of atropine (Table 1).

**Table 1:** The proportion of patients treated with stepwise concentrations of atropine.

	Atropine	Year										
		Baseline	1st	2nd	3rd	4th	5th	6th	7th	8th	9th	10th
Lower		**23 (100%)**	**23 (100%)**	**20 (87%)**	**18 (78%)**	**14 (61%)**	**13 (57%)**	**13 (57%)**	**13 (57%)**	**15 (65%)**	**15 (65%)**	**15 (65%)**
	0.05%	23 (100%)	15 (65%)	8 (35%)	7 (30%)	6 (26%)	6 (26%)	7 (30%)	8 (35%)	10 (43%)	11 (48%)	11 (48%)
	0.1%	0 (0%)	8 (35%)	12 (52%)	11 (48%)	8 (35%)	7 (30%)	6 (26%)	5 (22%)	5 (22%)	4 (17%)	4 (17%)
Higher		**0 (0%)**	**0 (0%)**	**3 (13%)**	**5 (22%)**	**9 (39%)**	**10 (43%)**	**10 (43%)**	**10 (43%)**	**8 (35%)**	**8 (35%)**	**8 (35%)**
	0.25%	0 (0%)	0 (0%)	3 (13%)	5 (22%)	9 (39%)	9 (39%)	9 (39%)	9 (39%)	7 (30%)	6 (26%)	6 (26%)
	0.5%	0 (0%)	0 (0%)	0 (0%)	0 (0%)	0 (0%)	1 (4%)	1 (4%)	1 (4%)	1 (4%)	2 (9%)	2 (9%)

### Changes in spherical equivalent

**Figure. 3:**
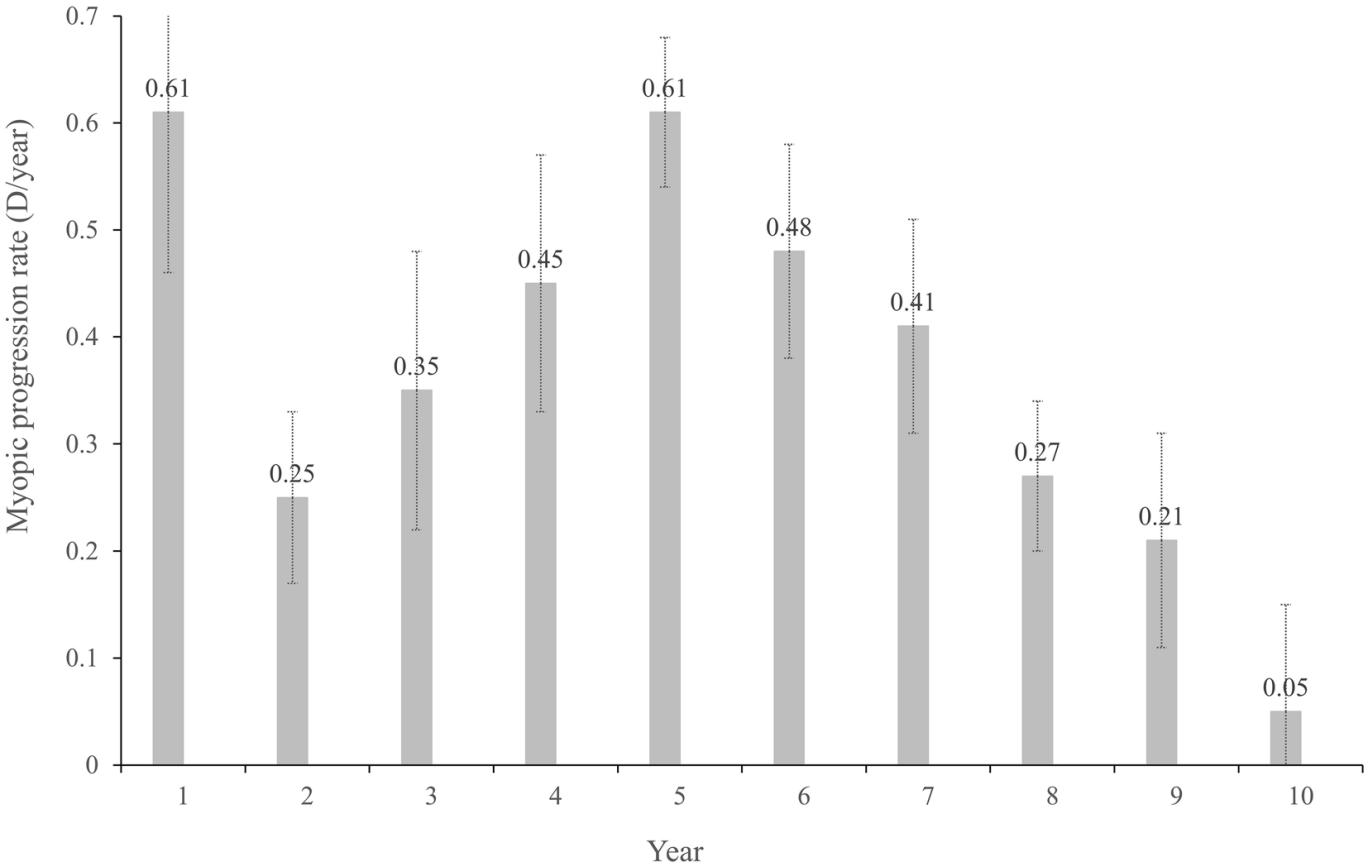
Mean annual myopic progression rate with stepwise concentrations of atropine treatment. D, diopter. Bars, standard error of the mean.

The mean progression of SE was − 0.30 ± 0.27 D/ year. During the 10-year-period, the maximum mean annual progression rate was − 0.61 ± 0.73 D/year in the 1st and 5th year, and the minimum was − 0.05 ± 0.34 D/year in the 10th year (Fig. 3).

### Characteristics of subjects and prediction of myopic progression

The characteristics of the subjects, initial age, sex, initial SE (grouping as ≤  − 1.0 D and >  − 1.0D) and the need of higher concentration of atropine treatment were analyzed to evaluate their effects on predicting the rate of myopic progression. Based on multivariate mixed-effect regression model analysis, younger initial age, female, higher initial SE and the need of higher concentration of atropine were related with faster myopic progression (*p* = 0.013, 0.017, 0.024 and 0.014; beta coefficient = 0.106, − 0.210, 0.073, and 0.293, respectively).

### Concentration of atropine versus myopic progression

**Figure 4:**
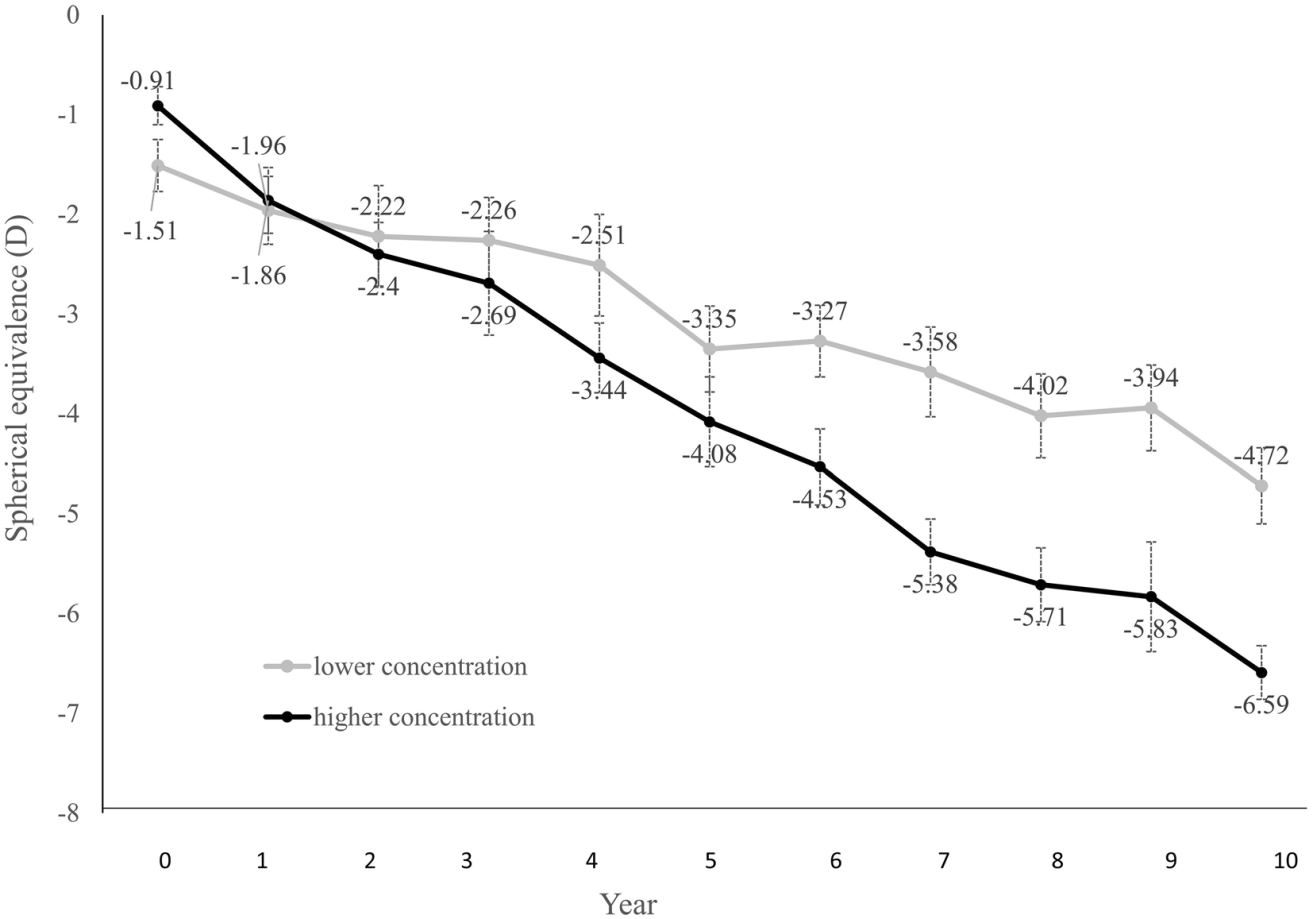
Spherical equivalence of stepwise lower concentration and stepwise higher concentration groups. D, diopter. Bars, standard error of the mean.

The subjects were divided into two groups; one with a stepwise lower concentration of atropine (≤ 0.1%, n = 13), while the other group had a stepwise higher concentration group (> 0.1%, n = 10) during the 10-year course. The male to female ratio in the stepwise lower and high concentration group were 10:3 and 4:6, accordingly. The stepwise lower concentration had a mean initial age of 7.31 ± 0.85 year-old, as for the higher group was 6.50 ± 1.18 year-old. The initial SE was − 1.51 ± 0.92 and − 0.91 ± 0.60 D in the stepwise lower and higher concentration group, separately. These baseline characteristics of the two groups had no significant difference (*p* = 0.07, 0.086, 0.09.) The myopic progression rate was slower in the stepwise lower concentration group with a mean progression rate of − 0.28 ± 0.43 D/year, and the higher-concentration group had an outcome of − 0.54 ± 0.58 D/year (*p* < 0.001) (Fig. 4). By the end of our study, 13 cases (56.5%) were ended up as high myopia (SE ≤  − 5.0D,) and 15 cases (65.2%) had less than − 5.0D of myopic progression among the 10-year follow-up period. Further eyecare habits, annual refraction and dilated detail fundus examination were strongly suggested for them.

## Discussion

Several studies have been performed to evaluate myopic progression under low concentration atropine in the past few years^20^. To our best of our knowledge, this study is the first long-term, throughout childhood and adolescents, up to 10 years study about how low concentration atropine works on myopic control in rea-world. The overall mean progression rate of myopia in this study was − 0.30 ± 0.27 D/year, which supports the effect of myopic control by long-term, low concentration atropine use. The subgroup analysis revealed that subjects in the stepwise lower concentration atropine group (≤ 0.1%) tended to have better control of myopia than those who required higher concentrations of atropine during the follow-up period. In other words, to achieve a more complete control of myopic progression, additional management or alternative treatment may be necessary.

In our study, the mean initial age was 6.96 year-old, younger than typical onset of myopia. In Taiwan, by 6 year-old, the prevalence of myopia in preschool children was 12.2%, higher than the other countries^21^. The myopic prevalence of 7-year-old school children increased to 25.41% in 2017^22^. Almost all the myopic population in this age was referred to ophthalmologist for medical intervention in Taiwan^23^.

**Figure 5:**
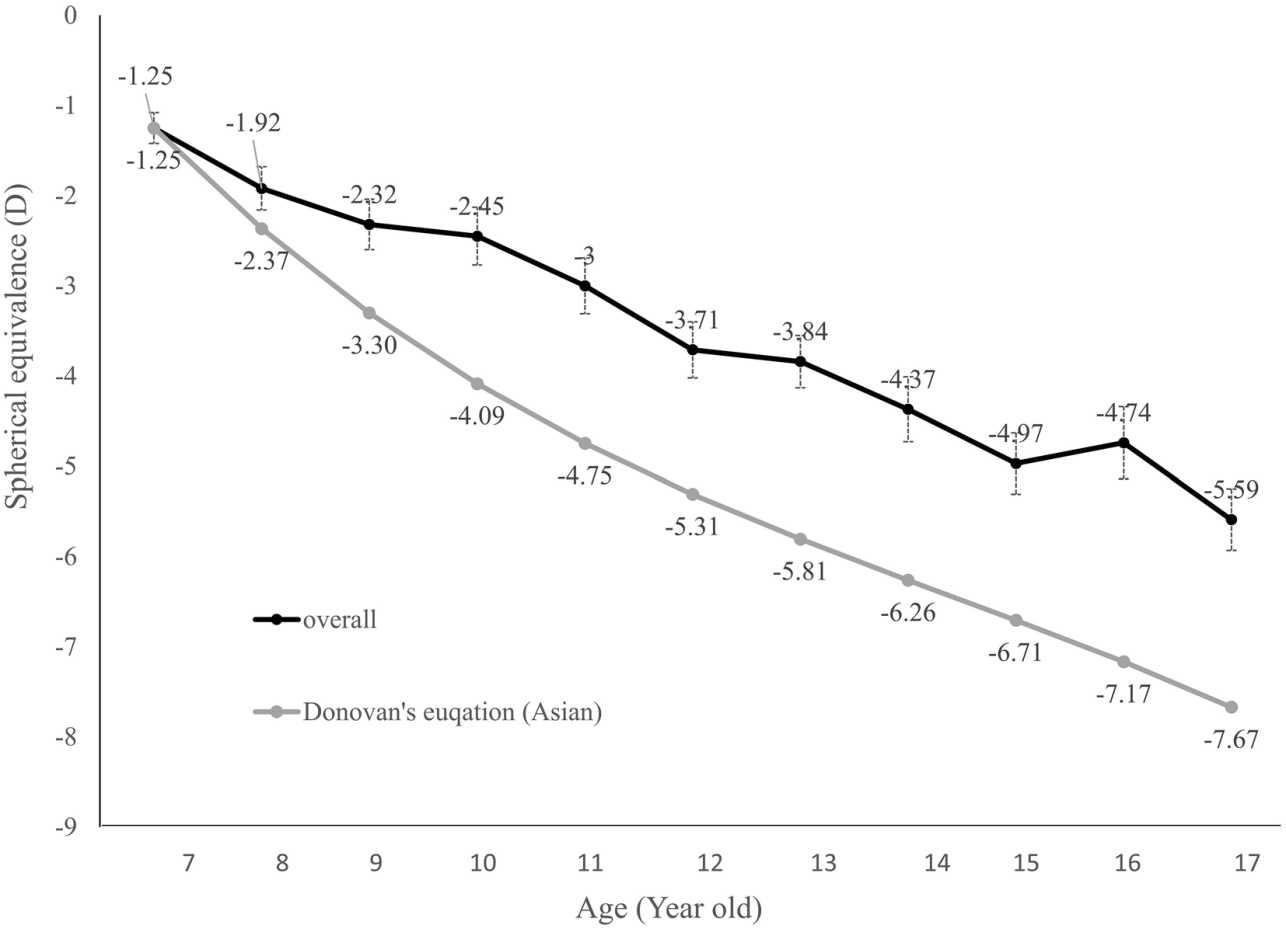
Spherical equivalence status in the patients with atropine treatment in our study and the estimated controls by predicted equation for 10 years. D, diopter. Bars, standard error of the mean.

Emmetropization is a concept that was introduced in 1997^24^. With the development of ocular refractive status, infants, who generally have hyperopic eyes, gradually evolve into emmetropia or stop at mild hyperopia^25^. Nonetheless, patients who develop myopia experience not only persistent changes in refractive status as they grow up but also structural changes, such as elongation of axial length. Generally, the rate of myopic progression decelerates with aging^26^. The results of this study showed a similar trend of myopia progression in the 10-year period, but the annular SE change and final SE of our study with stepwise low concentration of atropine treatment were less than the results from the predicting equation without treatment from Donovan et al.^26^, (Fig. 5). In the end of 10 years follow-up, the estimated SE would be − 7.67D without treatment compared to the mean of − 5.59D in our subjects treated with atropine eye drops. In our study, the myopic progression rate was higher around the 5th year. We presumed that increased near works from junior high school with higher academic stress might contribute to this result. Meanwhile, even though we did not have a control group, the results of this study were quite promising when weighing between previous studies. The myopic progression rate varies between culture and ethnicities. While the annual progression rate of myopia for Caucasians was − 0.55 D, Asians had a result of − 0.82 D. In the ATOM 1 study performed in Singapore, the placebo group followed for two years reported a myopic progression rate of − 1.20 ± 0.9 Din 2 years^27^. Another two-year study conducted in Taiwan showed that the rate of the control group was − 1.06 ± 0.61 D/year^28^. But in this study, the mean progression rate was − 0.30 ± 0.27 D/ year, which reached a more than 50% reduction compared to the outcomes above. According to a previous survey, with a more than 33% reduction in myopic progression, the number of high myopias could be decreased by as much as 75%^29^. In this study, the findings showed that atropine could effectively slow down the rate of myopic progression. The result supported that the stepwise method is effective in long-term myopic control and may even lower the risk of developing high myopia^30^.

The characteristics of subjects seem to play a role in determining the rate of myopic progression. A meta-analysis in 2012 reported that females tend to have faster myopic progression as well as younger age at baseline^26^. The Correction of Myopia Evaluation Trial (COMET) study revealed that patients with younger age at initial and higher initial SEs were more likely to have greater myopic progression^31,32^. In this study, female, younger initial age and higher initial SE had a faster myopic progression. The result is compatible to the recent study, that younger children tend to have poor response to low-concentration of atropine while comparing to older children^33^. To prevent the occurrence of high myopia, early intervention may be beneficial. According to a previous study performed on premyopic children, 0.025% atropine can prevent the onset of myopia^34^. Further studies can be applied to evaluate the low concentration stepwise strategy in such cases.

In a previous study, they found that pediatric ophthalmologists worldwide have very different practice patterns when involved in myopic control^35^. It could affect the efficacy of decreasing myopic progression. Thus, determining whose myopia can be properly managed by atropine alone and the timing for applying additional management may change their idea about coping with myopia. Previous studies found that the response rate of atropine varied. Rapid progression defined as > 1 D/year was found in 33% of children who received 0.1% atropine^28^. The ATOM 2 study applied a lower concentration of atropine in the two-year trial. However, in the 0.01%, 0.1%, and 0.5% groups, 9.3%, 6.4%, and 4.3% of subjects still had poor responses to atropine, respectively^36^. Clark et al.^37^ noted that 9% of children who received 0.01% atropine had rapid progression. Since a stepwise strategy was adopted in this study, the concentration of atropine may reflect how these subjects responded to pharmacological management. Those who kept using lower concentration atropine (≤ 0.1%) represented the “good responders,” while the stepwise higher concentration group (> 0.1%), who adjusted the concentration of atropine because of a faster progression rate, more than 0.5D per six months, were considered as “poor responders.” In other words, to achieve better control of myopia, when children who receive stepwise lower concentration atropine management require more than 0.1% of atropine, alternative or combined management, for example, orthokeratology, may be considered. As for the point of time to stop or taper the dose of atropine, shared decision making with patients and parents could be adopted after high school since accommodation ability decreased with aging^38^.

With a 10-year regular follow-up, the strength of this study is that the clinical use of low concentration atropine resulted in a positive effect on myopic control throughout the childhood and teenage period. The stepwise strategy of atropine use is a feasible method for managing myopic progression. In addition, the result offers a possible cut-off value of stepwise atropine use, and additional management could be considered, according to the concentration that the patient requires. Moreover, during the study period, no significant side effects were reported. The safety concern of low concentration atropine in a 10-year period is minimal, according to the outcome of this study. This study has some limitations. The study group was relatively small, and we did not have a proper control group. Another limitation was the evaluation about other myopia control interventions, such as orthokeratology or combined treatment, since these modalities were not common at that time of patient recruitment. Owing to the nature of a retrospective study, the lack of complete parameters, such as axial length, is the other limitation of this study. In this study, the initial low concentration of atropine treatment was similar to screening for drug response. Those who had higher concentration later were considered as the poor responders. The difference from those who kept receiving lower concentration may be contributed from the pharmacogenetic or environmental factors. This heterogenetic problem should have been solved by increasing the sample size and using randomization strategy. Lacking details of the subjects made it difficult to elucidate the underlying reasons. Due to the limitations of this study, a further prospective large sample size study is warranted.

## Conclusion

The results of this study not only show the efficacy of stepwise low-concentration atropine treatment on myopic control, but also demonstrate the long-term effect of this pharmacological management. Children who have a poor response to lower concentration atropine treatment and require a concentration higher than 0.1% during the stepwise management may indicate that additional or alternative management could be considered to reach an ideal control of myopia.
